# An Australian longitudinal pilot study examining health determinants of cardiac outcomes 12 months post percutaneous coronary intervention

**DOI:** 10.1186/s12872-016-0203-9

**Published:** 2016-02-03

**Authors:** Karen-leigh Edward, John Stephenson, Jo-Ann Giandinoto, Andrew Wilson, Robert Whitbourn, Jack Gutman, Andrew Newcomb

**Affiliations:** Australian Catholic University and St Vincent’s Private Hospital Melbourne Ltd, Locked Bag 4115 MDC, Fitzroy, 3065 Australia; University of Huddersfield, Huddersfield, United Kingdom; University of Melbourne and St Vincent’s Hospital Melbourne, Melbourne, Australia

**Keywords:** Percutaneous coronary intervention, Wellbeing, Quality of life, Depression, Gender, Resilience

## Abstract

**Background:**

Percutaneous coronary intervention (PCI) is a very common revascularisation procedure for coronary artery disease (CAD). The purpose of this study was to evaluate cardiac outcomes, health related quality of life (HRQoL), resilience and adherence behaviours in patients who have undergone a PCI at two time points (6 and 12 months) following their procedure.

**Methods:**

A longitudinal pilot study was conducted to observe the cardiac outcomes across a cohort of patients who had undergone a percutaneous coronary intervention (PCI). Participants who had undergone PCI 6 months prior were invited. Those participants who met the inclusion criteria and provided consent then completed a telephone survey (time point 1). These participants were then contacted 6 months later (i.e. 12 months post-intervention, time point 2) and the measures were repeated.

**Results:**

All patients (n = 51) were recorded as being alive at time point 1. The multiple model indicated that controlling for other factors, gender was significantly associated with a linear combination of outcome measures (*p* = 0.004). The effect was moderate in magnitude (partial-η^2^ = 0.303), where males performed significantly better than females 6 months after the PCI procedure physically and with mood. Follow-up univariate ANOVAs indicated that gender differences were grounded in the scale measuring depression (PHQ9) (*p* = 0.005) and the physical component score of the short form measuring HRQoL (SF12-PCS) (*p* = 0.003). Thirteen patients were lost to follow-up between time points 1 and 2. One patient was confirmed to have passed away. The pattern of correlations between outcome measures at time point 2 revealed statistically significant negative correlation between the PHQ instrument and the resilience scale (CD-RISC) (*r* = -0.611; *p* < 0.001); and the physical component score of the SF-12 instrument (*r* = -0.437; *p* = 0.054).

**Conclusions:**

Men were performing better than women in the 6 months post-PCI, particularly in the areas of mood (depression) and physical health. This pilot results indicate gender-sensitive practices are recommended particularly up to 6 months post-PCI. Any gender differences observed at 6 month appear to disappear at 12 months post-PCI. Further research into the management of mood particularly for women post-PCI is warranted. A more detailed inquiry related to access/attendance to secondary prevention is also warranted.

## Background

Cardiovascular disease (CVD) has over the last decade emerged as the single most important cause for death worldwide [1]. Two recent international studies, the INTERHEART study in 2005 and the INTERSTROKE study in 2010 have confirmed that in both developing and developed countries, the risk factors for CVD include: smoking, hypertension, abdominal obesity, lack of physical activity, abnormal blood lipid profiles, diabetes, alcohol intake, psychosocial factors and a diet deficient in fruits, vegetables and fish [2, 3]. Percutaneous coronary intervention (PCI) is a very common revascularisation procedure for coronary artery disease (CAD), and indications for PCI include myocardial infarction (MI), stable and unstable angina pectoris (SAP and UAP respectively). In 2000, the age-standardised ratio of PCI procedures in males compared to females was around 3.1 to 1; this is despite males experiencing heart attacks at only twice the rate of females [4]. Irrespective of recent advances in surgical technology, PCI is associated with a range of adverse post-procedural complications, including acute bleeding, cardiac events, vascular complications and mortality [5–7].

A significant proportion of PCI patients also experience mental health issues, although the direction of this relationship is unclear. Anxiety and depression are prevalent in 25-50 % of CAD patients [8–10] and are associated with readmission for further cardiovascular morbidity [9, 11, 12], decreased health-related quality of life and mortality [10, 13]. There is evidence to suggest that levels of anxiety and depression in the majority of PCI patients remain relatively stable 12 months post-procedure, and baseline levels of anxiety and depression are associated with either symptom improvement or deterioration among those who experience changes to distress [14].

Research findings are inconclusive as to the impact of PCI on quality of life post-procedure. Some evidence indicates patients over the age of 70 years are more likely to experience deteriorating and poorer mental and physical health-related quality of life than those under 70 years at 36 months post-intervention, irrespective of adverse events during outcomes [15]. The impact of psychopathology such as anxiety and depression is known to increase risk of readmission [11] and mortality [13], but little is known about the efficacy of psychological interventions on long-term outcomes of PCI. The short term impacts (<6 months) of coronary care for patients indicates HRQoL concerns should not drive revascularisation decisions [16], that mood can predict worsening heart health [17, 18] and QoL should be considered in future research in order to inform practice decisions with this group of patients [19]. There appears to be no studies that have examined the notion of personal resilience in this group of patients.

The aim of this longitudinal prognostic study was to evaluate cardiac outcomes, HRQoL, personal resilience, mental health and adherence behaviours in patients who have had undergone a PCI measured at two time points (6 months and 12 months) post-intervention. The objectives of this study were to (1) inform clinical care practice of the care of patients who undergo PCI, and (2) provide pilot information of patients related to QoL, medication adherence behaviours, personal resilience and mental health post a PCI.

## Methods

### Research design

A longitudinal pilot study design was undertaken. Observational research, such as this study allowed the use of repeated measures of the same cohort over 12 months. The benefit of this type of study is the ability to assess the significance of predictors of outcomes post-PCI.

### Participants and recruitment

#### Sampling

The study aimed to recruit a total of 49 participants. As a pilot, the study was not powered to detect significant effects or was subject to a formal sample size calculation. However, a sample size was desired which would allow the assessment of incidence rates with a 95 % confidence interval with +/- 10 % precision (i.e. to have 95 % confidence that the estimated rate was within 10 % of its true value in the population) assuming a prevalence of hospital readmission of about 15 %. The sample size also provided sufficient power to show that the correlation between continuously distributed variables (adherence, QOL etc.) and was statistically significant if the correlation coefficient was ≥ 0.39 and provided sufficient power to test 3-4 variables in multiple regression models without compromising stability of estimates.

### Exclusion criteria

Participants were excluded from the study if they were under the age of 18 years, were unable to give informed consent or English was not their first language.

### Ethics, consent and permissions

Ethical clearance was obtained from both the hospital (St Vincent’s Hospital Melbourne) and university (Australian Catholic University) Human Research Ethical Committees (protocol numbers LRR 020/13 and 2014 75 V respectively). Informed verbal consent was obtained before data collection was undertaken. Consent to publish findings in aggregated formats was obtained in the consent process for all participants.

### Recruitment

Participants were identified via a database. This database is called The Victorian Cardiac Outcomes Registry (VCOR) and became operational in December 2012 as a tool to help improve safety and quality of healthcare provided to cardiovascular patients in the state of Victoria. The collection of data, coordinated by the Victorian Cardiac Clinical Network, and jointly funded by Victorian Department of Health and Medibank Private, invited all public and private health services that perform PCI to join VCOR. St Vincent’s Private Hospital Melbourne was the participating site in the original VCOR collection and ethics approval was obtained for the use of this database as a source of recruitment for our study. Participants were invited to participate consecutively using the database contact details and after providing informed consent by the research interviewer completing the questionnaire with participants, they were enrolled in the study. Recruitment took place between 1/2014 and 11/2014.

### Data collection

Data was collected via a telephone interview at both time points (6 months and 12 months post-PCI). To evaluate lifestyle cardiac risk factors, patients were asked about their tobacco use, physical activity, diet, cholesterol/lipids (total cholesterol above 5.5 mmol/L is considered high), and about any weight gain. Patients were also asked about their adherence to cardiac rehabilitation, and all cause readmissions. Mortality was recorded if this had occurred (see demographic information Table 1).

**Table 1:**
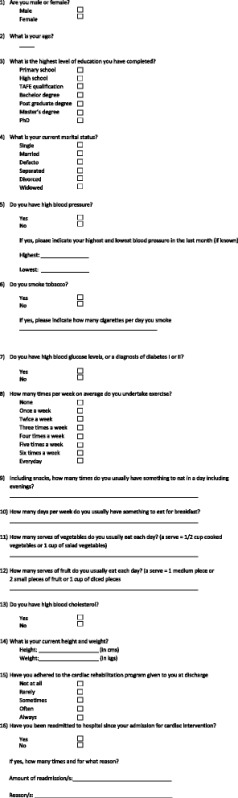
Demographics and Health Information

Adherence was measured using the Brief Medication Questionnaire (BMQ). This self-report tool for monitoring adherence is used to identify patients who need assistance with their medications, assessing patient concerns, and evaluating new programs. The tool includes a 5-item Regimen Screen that asks patients how they took each medication in the past week, a 2-item Belief Screen that asks about drug effects and bothersome features, and a 2-item Recall Screen about potential difficulties remembering. Validity was assessed in 20 patients using the Medication Events Monitoring System (MEMS). Results varied by type of non-adherence, with the Regimen and Belief Screens having 80–100 % sensitivity for “repeat” non-adherence and the Recall Screen having 90 % sensitivity for “sporadic” non-adherence. The BMQ appears more sensitive than existing tools and may be useful in identifying and diagnosing adherence problems [20].

Depression was evaluated using the Patient Health Questionnaire (PHQ9) [21]. The sensitivity and specificity of the PHQ9 is 83 and 92 % respectively if the scaled response is requested as in the current investigation (i.e. not at all = 0, several days = 1, more than half the days = 2 and nearly every day = 3). The PHQ is positive if the participant scores 3 or more points.., Minor depression was defined to be given by a score between 5 and 9, and severe depression was defined to be given by a score of 20 or more.

To measure resilience, the Connor-Davidson resilience scale (CD-RISC) was used. The original CD-RISC is a 25-item scale assessing resilience during the last month, with higher scores indicating higher resilience capacity [22]. Each item is rated on a 5-point range of responses from not true at all (0) to true nearly all time (4). The total score ranges from 0–100. The CD-RISC has been tested in the general population, as well as in clinical samples, and demonstrates excellent psychometric properties, with good internal consistency and test–retest reliability. The scale exhibits validity relative to other measures of resilience. The CD-RISC is a brief, self-rated measure of resilience that can measure resilience in the context of health status.

The SF-12 was used to evaluate health related quality of life. The SF-12 comprises: two questions about physical functioning; two questions on role limitations because of physical health problems; one question about bodily pain; one question related to general health perceptions; one question on vitality, energy/fatigue; one question on social functioning; two questions about role limitations because of emotional problems; and two questions on general mental health. The 12 items on the SF-12 are summarized in two weighted summary scales - mental health score (MCS) and physical health score (PCS) - where lower scores indicate more severe disability [23]. The SF12 - PCS and MCS component summary scales – were scored using a norm-based method. Regression weight for the PCS and the MCS are derived from the United States of America population [24].

### Data analysis

The sample was summarised descriptively. The following outcome measures were considered: resilience (as measured by the Connor-Davidson resilience scale (CD-RISC)) [22]; depression (as measured by the Patient Health Questionnaire (PQ-9)) [21]; adherence (as measured by the Brief Medication Questionnaire (BMQ)) [20] and quality of life (as measured by the MCS and PCS sub-scales of the SF-12 questionnaire) [23]. The following variables collected on patients were initially considered for their association with the outcome measures at each time point: gender, age, hypertension, tobacco use, raised blood glucose (diabetes), physical activity, diet, cholesterol/lipid levels, and weight. Additional demographic and health-related variables were collected for descriptive analysis. The survival status of each patient (i.e. alive or dead) was also recorded at each time point.

Exploratory analyses were conducted on the data to assess the extent of missing data, frequencies of groups in categorical variables, possible collinearity between predictor variables and suitability of the outcome measures for a multivariate treatment. Categorical variables with a high number of categories including low-frequency categories were combined appropriately for analysis to ensure that each category had a sufficient number of individuals for analysis. Patterns of correlations between outcome variables were assessed for suitability for multivariate treatment.

Analysis of variance (ANOVA) and multivariate analysis of variance (MANOVA) were conducted on data collected at 6 months after the PCI procedure (time point 1) and 12 months after the PCI procedure (time point 2), with the outcome measures included in the MANOVA procedure being those found to be substantively correlated. To avoid over-fitting the models, a modelling strategy was derived whereby each predictor variable was initially assessed in a series of uni-variable models. Variables appearing to be important at this stage were then carried forward for inclusion in multiple models, with standard statistics reported from these models. In the event of a particular predictor variable being significantly related to the linear combination of outcome measures, follow-up univariate analyses of variance (ANOVAs) were conducted to investigate the sources of such associations.

A series of doubly multivariate repeated measures analyses of variance was also conducted on the data to further investigate the effect of the time component. A similar modelling strategy was implemented as for the ANOVA/MANOVA models; with between-subjects variables initially assessed on a univariable basis within these models; any variables found to be of substantive significance were carried forward for inclusion in a multiple model.

## Results

### Descriptive and exploratory analysis

Data was collected on 51 patients for this pilot study. Baseline sample characteristics are summarised descriptively in Table 2.

**Table 2:** summary of baseline sample characteristics

Categorical factors	Frequency (valid %)
Gender	
Male	41 (80.4 %)
Female	10 (19.6 %)
Highest level of qualification	
Primary school	2 (3.9 %)
High school	33 (64.7 %)
TAFE qualification	13 (25.5 %)
Postgraduate degree	3 (5.9 %)
Marital status	
Single	3 (5.9 %)
Married	37 (72.5 %)
De facto	4 (7.8 %)
Separated	1 (2.0 %)
Divorced	1 (2.0 %)
Widowed	5 (9.8 %)
Hypertension	
Yes	30(58.8 %)
No	21 (41.2 %)
Smoking status	
Smoker	1 (2.0 %)
Non-smoker	50 (98.0 %)
Diabetes	
Yes	12 (23.5 %)
No	39 (76.5 %)
Exercise frequency	
None	3 (5.9 %)
1-3 times per week	14 (27.5 %)
4-6 times per week	6 (11.8 %)
Daily	28 (54.9 %)
Cholesterol level (total above 5.5 mmol/L)	
High	35 (70.0 %)
Normal	15 (30.0 %)
Adherence to cardiac rehabilitation	
Not at all	18 (36.7 %)
Sometimes	6 (12.2 %)
Often	8 (16.3 %)
Always	17 (34.7 %)
Readmission post intervention	
Yes	16 (32.0 %)
No	34 (68.0 %)
Numerical covariates	Mean (SD)
Age	72.3 (8.82)
Height (cm)	173.9 (8.34)
Weight (kg)	82.7 (14.0)

Exploratory analyses identified a small amount of missing data on certain predictor variables. The total amount of missing data was less than 0.5 %. Missing data on predictor variables only was imputed using expectation maximisation.

Only one participant self-reported as a user of tobacco. This variable was not considered further. No evidence for collinearity was observed between the remaining independent variables considered for analysis.

### Analysis – time point 1 (6 months post PCI)

**Fig. 1:**
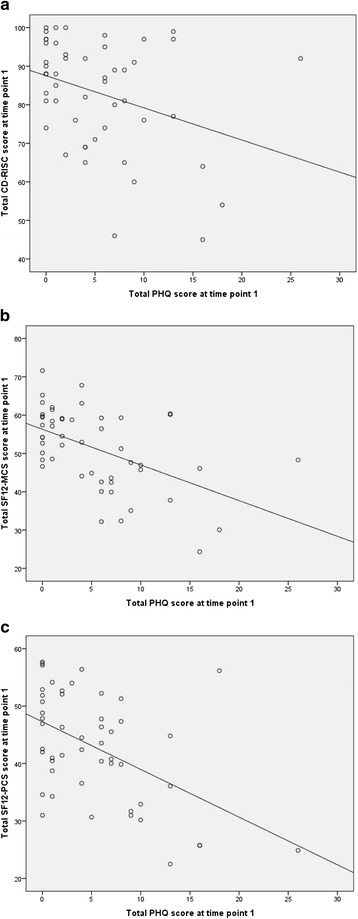
**a**: Correlation between CD-RISC scores and PHQ scores at time point 1. **b**: Correlation between SF12-MCS scores and PHQ scores at time point 1. **c**: Correlation between SF12-PCS scores and PHQ scores at time point 1

All patients were recorded as being alive at time point 1. The pattern of correlations between outcome measures at time point 1 revealed statistically significant moderate negative correlation between the PQ-9 instrument and: the CD-RISC instrument (*r* = -0.331; *p* = 0.018); the mental component summary (MCS) subscale of the SF-12 instrument (*r* = -0.517; *p* < 0.001); and the physical component summary (PCS) subscale of the SF-12 instrument (*r* = -0.506; *p* < 0.001); implying that a multivariate treatment was appropriate for these outcome measures. The correlations are illustrated in Fig. 1 (a)–(c).

No significant or substantive correlation was recorded between the BMQ instrument and the other outcome measures. Hence a separate univariate analysis was conducted on this outcome.

Univariable models indicated that gender, exercise levels, cholesterol/lipid levels and weight exhibited at least some substantively important association with a linear combination of the PQ-9, CD-RISC, SF12-MCS and SF12-PCS scales. These variables were carried forward to a multiple multivariate model including these outcome measures. The variables age, education, hypertension, diabetes status, frequency of eating, and post-intervention readmission did not appear to be important in univariable models and were not considered further with respect to these outcome measures.

The multiple model indicated that controlling for other factors, gender was significantly associated with a linear combination of outcome measures (Λ = 0.697; F_4,40_ = 4.45; *p* = 0.004). The effect was moderate in magnitude (partial-η^2^ = 0.303). Follow-up univariate ANOVAs indicated that gender differences were grounded in the PQ-9 scale (F_1,44_ = 8.85; *p* = 0.005) and the SF12-PCS scale (F_1,44_ = 10.2; *p* = 0.003). On the PHQ scale, the mean male score was 13.1 (SD 5.21); the mean female score was 17.1 (SD 3.57). On the SF12-PCS scales, the mean male score was 53.1 (SD 9.58); the mean female score was 44.9 (SD 11.8). Hence males performed significantly better than females on both these scales 6 months after the PCI procedure.

No other factor was found to be statistically significant in the multiple model; however, some of the remaining factors indicated a degree of substantive significance.

None of the categorical factors or covariates considered exhibited any statistically significant or substantive association with the BMQ outcome measure.

### Analysis – time point 2 (12 months post PCI)

Thirteen patients were lost to follow-up between time points 1 and 2. One patient was confirmed to have passed away. Twelve patients refused to participate further in the study or did not respond to requests; of these, 10 were known to be alive at time point 2, with the status of two patients being uncertain.

The pattern of correlations between outcome measures at time point 2 revealed statistically significant or near-significant negative correlation between the PHQ instrument and the CD-RISC instrument (*r* = -0.611; *p* < 0.001); and the PCS subscale of the SF-12 instrument (*r* = -0.437; *p* = 0.054). Most other correlations between outcome measures, were also negative and moderate in magnitude; implying that a multivariate treatment was appropriate for all outcome measures, including the BMQ outcome. Univariable models indicated that education level and hypertension exhibited at least some substantively important association with a linear combination of the PHQ, CD-RISC, BMQ, SF12-MCS and SF12-PCs scales. These variables were carried forward to a multiple model. This model did not indicate either education level or hypertension to be statistically associated with a linear combination of the outcome measures.

### Repeated measures analysis

Outcome measured recorded at time points 1 and 2 are summarised in Table 3 below.

**Table 3:** summary of outcome measure scores at time points 1 and 2

Measure	Time point 1 (mean (SD)) – all patients	Time point 2 (mean (SD)) – all patients	*p*-value for change (calculated from patients providing values at both time points)
CD-RISC	83.3. (14.0)	80.6 (17.1)	0.083
PQ-9	13.9 (5.16)	4.97 (5.26)	<0.001
BMQ	1.59 (0.876)	2.78 (2.24)	0.022
SF12-MCS	43.0 (9.57)	57.0 (8.28)	<0.001
SF12-PCS	51.4 (10.5)	39.7 (10.8)	0.004

Hence a substantive change was recorded in all measures except the CD-RISC measure between the two time points. The reduction in PQ-9 scores, and the increase in SF12-MCS scores represent improvements; the increase in BMQ scores and the reduction in SF12-PCS scores represent deteriorations. A null model with no between-subjects factors or covariates indicated that time point 2 (12-month) scores were significantly different from time point 1 (6-month scores) on the PHQ outcome (F_1,18_ = 143.7; *p* < 0.001), the MCS outcome (F_1,18_ = 24.5; *p* < 0.001), the PCS outcome (F_1,18_ = 11.0; *p* = 0.004) and the BMQ outcome (F_1,18_ = 6.33; *p* = 0.022). Scores on the CD-RISC outcome were not significantly different between time points 1 and 2. No between-measures factors or covariates measured in univariable models were statistically significant or exhibited substantive importance.

## Discussion

The analysis reveals that men are performing better than women 6 months after the PCI procedure on a range of outcome measures, particularly depression (as measured by the PQ-9 instrument) and physical health (as measured by the SF-12-PCS instrument). Using the PHQ09 improvements in mood particularly depression has been associated with improved adherence in cardiac inpatient cardiac units who were hospitalized for acute coronary syndrome, heart failure, or arrhythmia in the 6 months following hospitalisation [25] however, assessment of gender differences requires further inquiry since the enrolment of women in heart studies remains low. In our study, much of these gender differences have disappeared by 12 months. Furthermore, no other grouping variable discriminates between patients at either 6 or 12 months after PCI. This may be because as a pilot study, the study is underpowered to detect any such effects that may exist; in particular the analysis of time point 2 data, which was subject to heavy attrition loss.

While significant changes in most outcomes were recorded between the two time points, the overall pattern of improvement is not clear; with the direction of movement being positive for two measures and negative for two measures. Improvement or deterioration is not more noticeable in any sub-group than any other. Again, this may be ascribed to a loss of power as a result of the low sample size. It is also possible that attrition bias was introduced by the loss of 25.5 % of the sample between 6 and 12 months.

Mood and physical health considerations particularly for women patients in the first 6 months post-PCI appears an area for further investigation. It is known that within Australia, data that specifically addresses women and CHD is limited [26–28], even in the knowledge that the traditional risk factors for CVD in women is increasing. Clinically women can present with a wider range of symptoms, which can lead to delayed diagnosis should problems arise post-intervention. In addition, psychosocial factors and conventional CVD risk factors often coexist (i.e. depression and heart disease). Mental health problems such as depression give rise to perceived reduced social support, and can negatively impact of recovery post PCI. Depression can also become chronic over time. Care planning related to prodromal symptoms and devising a plan of action in collaboration with patients post PCI is recommended. In addition, assessment of existing and/or required social supports and pre-existing mental health problems is recommended to ensure access to required supports post-PCI for patients in a timely manner.

### Limitations of the study

The longitudinal nature of this study, whilst having an advantage of observing and controlling for differences in the same cohort of people over a period of time when compared to cross-sectional study, does have some limitations. Data collection in this study was time-consuming and resulted in a reduced number of participants completing surveys at the 12-month time point of the study, with the resulting smaller sample size at this time point leading to a reduction in power of analyses conducted at this time point. The sample size was small, with a small number of women being represented in the cohort; therefore interpretations of the findings are to be considered conservatively in the context of the pilot study setting. Another limitation of this study was the use of self-report for smoking status and in surveys: although each of the measures used have been tested for reliability and validity, the nature of surveys relies on participants’ ability to recall information, answer truthfully, understand the questions and scales used; and the surveys must have the ability to remove potential for response bias (i.e. participants will answer questions in a certain way regardless of the question being asked). In this study questionnaires were completed over the telephone with a researcher. Future research may consider providing participants with a paper/online copy of the questionnaire where they would be able to answer the questions anonymously.

## Conclusions

This pilot study has provided insight into the potential social and physical impacts post-PCI, particularly for women patients with regard to mood and physical health with men performing better physically and with mood 6 months post PCI. In consideration of the differences in symptom presentation between men and women undergoing cardiac care, interventions need to be tailored in an individual and gender sensitive manner following PCI. Given the paucity of research related to women following PCI further research into the management of mood particularly for women is warranted. More detailed inquiry related to access/attendance to secondary prevention is also warranted.

## Abbreviations

ANOVA: analysis of variance
BMQ: brief medication questionnaire
CAD: coronary artery disease
CD-RISC: Connor-Davidson resilience scale
CVD: cardiovascular disease
HRQoL: health related quality of life
MANOVA: multivariate analysis of variance
MCS: mental component summary
MI: myocardial infarction
PCI: percutaneous coronary intervention
PCS: physical component summary
PHQ: patient health questionnaire
QOL: quality of life
SF12: short form 12
STEMI: ST-elevation myocardial infarction
VCOR: Victorian Cardiac Outcomes Registry
